# Identification of Noninvasive Biomarkers for Gallbladder Carcinoma

**DOI:** 10.7759/cureus.117887

**Published:** 2026-10-13

**Authors:** Aman Gupta, Tarun Kalra, Varsha Kumar, Girijesh K Patel, Sanjay Singh

**Affiliations:** 1 General Surgery, Moti Lal Nehru Medical College, Prayagraj, IND; 2 Gastrointestinal Surgery, Moti Lal Nehru Medical College, Prayagraj, IND; 3 Pathology, Moti Lal Nehru Medical College, Prayagraj, IND; 4 Biotechnology, Motilal Nehru National Institute of Technology, Prayagraj, IND

**Keywords:** biomarkers, cancer antigen 125 (ca-125), carbohydrate antigen 19-9 (ca 19-9), carcinoembryonic antigen (cea), carcinoma gallbladder, early diagnosis, gallstones, mirna-155, mirna-21, roc analysis

## Abstract

Background

Carcinoma of the gallbladder (GBC) is an aggressive malignancy of the hepatobiliary system with a poor prognosis due to late presentation and a lack of effective diagnostic tools. Conventional imaging often fails to accurately detect the disease in its early stages. Both abdominal USG and contrast-enhanced computed tomography (CECT) have lower sensitivities for early-stage GBC. This highlights the need for reliable noninvasive biomarkers that may facilitate early diagnosis and improve clinical outcomes. These novel molecular markers may be used alone or in combination with serum tumor markers to enhance their diagnostic utility. This study was performed to identify novel, noninvasive biomarkers for the diagnosis of GBC and determine their accuracy in differentiating carcinoma from other benign pathologies of the gallbladder.

Materials and methods

This prospective observational case-control study was conducted over a period of one year, from April 2025 to March 2026, in the Department of General Surgery, Motilal Nehru Medical College, Prayagraj. A total of 60 patients were included, comprising 30 histologically confirmed GBC cases and 30 controls with benign gallbladder disease. Detailed clinical evaluations, laboratory investigations, and imaging studies were performed. Blood samples were collected prior to intervention, and biomarkers, including carbohydrate antigen (CA) 19-9, carcinoembryonic antigen (CEA), and CA-125, were analyzed using ELISA, while microRNA (miRNA)-21 and miRNA-155 were analyzed using quantitative reverse transcription-polymerase chain reaction (qRT-PCR) techniques. Statistical analysis was performed, and diagnostic accuracy was assessed using receiver operating characteristic (ROC) curve analysis.

Results

Patients with GBC showed significantly higher levels of the tumor markers CA 19-9, CEA, and CA-125 compared to controls (p < 0.001). The molecular biomarkers miRNA-21 and miRNA-155 were also markedly elevated in carcinoma cases and strongly correlated with tumor stage and histological grade. The combination of biomarkers (CA 19-9 and miRNA-21) showed superior diagnostic accuracy (91.7%), with higher sensitivity (93.3%) and specificity (90%) on ROC analysis (area under the curve (AUC) of 0.956). Clinical and radiological findings were consistent with advanced disease in most cases.

Conclusion

Noninvasive biomarkers, particularly when used in combination, demonstrate high diagnostic accuracy for the early detection of GBC. Integration of biomarker analysis with clinical and radiological evaluation can further improve diagnostic utility and facilitate better clinical management. These findings support the potential role of biomarker-based screening strategies in high-risk populations.

## Introduction

Carcinoma of the gallbladder (GBC) is a silent and rapidly progressive tumour, often with fatal outcomes. In the Indian population, the majority of cases (>80%) are diagnosed at an advanced stage, and only 10% of cases are suitable for upfront surgery at initial presentation [1]. This contributes to its poor long-term survival (5-year survival rate of <20%) [2]. Because surgical resection is the only potentially curative treatment, there is an urgent need for reliable non-invasive tools that can distinguish malignant from benign gallbladder conditions at an early, potentially curable stage. Conventional serum tumour markers, such as carcinoembryonic antigen (CEA) and carbohydrate antigen (CA) 19-9, have limited sensitivity for small mucosal lesions (14-50%) [3] and for early malignant transformation in the context of gallstones or cholecystitis, resulting in a clear diagnostic gap that non-invasive biomarkers could fill. Non-invasive biomarkers have the potential to change the diagnostic landscape for gallbladder carcinoma by improving early detection, guiding triage for further imaging or early referral to specialized centres, and allowing better stratification for surgery. While traditional markers (CA 19-9, CEA) retain value as adjuncts, novel modalities, especially circulating miRNA/exosomal signatures and liquid biopsy approaches, are the most promising frontiers in current research [4].

Therefore, systematic evaluation of selected non-invasive biomarkers in well-characterized cohorts of patients with GBC and appropriate controls is warranted. Such studies should (a) quantify biomarker differences between malignant and benign disease, (b) determine diagnostic accuracy, and (c) assess whether combinations of biomarkers improve diagnostic performance beyond single analytes. This approach can identify diagnostic cut-offs for promising non-invasive markers that can be prospectively validated in larger cohorts in the future. The present study aims to contribute to that evidence base by evaluating the diagnostic performance of selected non-invasive biomarkers that are practical, reproducible, and clinically relevant.

## Materials and methods

Study design and setting

This prospective case-control study was conducted in the Department of General Surgery at Swaroop Rani Nehru Hospital, Motilal Nehru Medical College, Prayagraj, over a period of one year, from April 2025 to March 2026, as per the protocol approved by the Institutional Ethics Committee (IEC) (Ref. No. ECR/922/INST/UP/RR-22) on August 19, 2025.

Study population

The study included patients aged 18 years and above with a clinical, radiological, and histopathological diagnosis of GBC. The control group consisted of age- and sex-matched patients diagnosed with benign gallbladder disease, such as cholelithiasis without malignancy. Patients with acute cholecystitis were not included.

Inclusion criteria

Patients aged 18 years and above with a clinical, radiological, and histopathological diagnosis of GBC were included as cases. Patients with cholelithiasis without malignancy were included as controls. Written informed consent was obtained from all participants prior to inclusion.

Exclusion criteria

Patients with other malignancies, recent infections, inflammatory or autoimmune disorders, those receiving immunosuppressive therapy, and those who did not provide consent were excluded from the study.

Sample size calculation

Using an expected sensitivity of 80% for miRNA-21 in biliary tract cancers [5], the sample size (n) was calculated using the formula:

n = *Z&sup2; &times; p(1 &minus; p)/d&sup2;*

where Z = 1.96 for a 95% CI, p is the expected sensitivity (0.80), and d = 0.10 (absolute precision). The calculated sample size was 60.4, approximated to 60. Accordingly, 30 cases and 30 controls were included in the study.

Sampling technique

A consecutive sampling technique was used to recruit eligible patients during the study period.

Study variables

The study included demographic (age, sex, residence, and socioeconomic status), clinical (symptoms and signs), radiological (USG and CT findings), laboratory (routine haematological and biochemical tests and conventional tumour markers), and molecular variables. Tumour staging was performed according to the Tumour, Node, Metastasis (TNM) classification of the American Joint Committee on Cancer (AJCC), 8th edition.

Study procedure

After obtaining institutional ethical clearance and informed consent, eligible patients were enrolled in the study. A detailed clinical history was obtained, and a physical examination was performed for all participants. Data were recorded in a structured case record form.

Radiological evaluation, including USG and CECT of the abdomen, was performed for the assessment of gallbladder pathology and staging. The scans were performed before biochemical tests and were interpreted by radiologists who were blinded to the biomarker results. Blood samples (10 mL) were collected from all participants under aseptic conditions prior to any surgical or chemotherapeutic intervention. After allowing the blood to coagulate for 20 minutes, serum was separated by centrifugation at 2,000 × g for 10 minutes. The cleared serum samples were aliquoted into 500 µL tubes and stored at -80°C until further analysis.

Tumour markers, including CA 19-9, CEA, and CA-125, were assayed using ELISA techniques, while microRNAs, such as miRNA-21 and miRNA-155, were analysed using qRT-PCR on serum samples.

Gallbladder tissue samples were also collected postoperatively from cases and preserved in RNAlater solution (Cat. #AM7020, Invitrogen, Vilnius, Lithuania) overnight and then stored in an ultra-low-temperature freezer at −80°C until further processing at Motilal Nehru National Institute of Technology (MNNIT), Prayagraj.

Serum miRNA was isolated using a serum/plasma miRNA isolation kit (Qiagen, Cat. #217184) and quantified using a NanoDrop 2000 (Thermo Scientific, MA, USA). A total of 100 ng of RNA was used for cDNA synthesis using the ABScript miRNA First-Strand Synthesis Kit (ABclonal, Woburn, MA, USA; Cat. No. RK30170), with specific RT primers for U6, miRNA-21, and miRNA-155 listed in Table 1.

**Table 1:** List of primers used for qRT-PCR. miR: microRNA; RT: Reverse transcription; qRT-PCR: Quantitative reverse transcription-polymerase chain reaction.

Primer name	Primer sequence (5′-3′)
U6 RT primer	AAAATATGGAACGCTTCACGAATTTG
U6 forward primer	CTCGCTTCGGCAGCACATATACT
U6 reverse primer	ACGCTTCACGAATTTGCGTGTC
miR-21 RT primer	GTCGTATCCAGTGCAGGGTCCGAGGTATTCGCACTGGATACGACTCAACA
miR-21 forward primer	TCGGCGTAGCTTATCAGACTGA
miR-21 reverse primer	GTCGTATCCAGTGCAGGGTCCGAGGT
miR-155 RT primer	GTCGTATCCAGTGCAGGGTCCGAGGTATTCGCACTGGATACGACACCCCT
miR-155 forward primer	TCGGCGTTAATGCTAATCGTGAT
miR-155 reverse primer	GTCGTATCCAGTGCAGGGTCCGAGGT

miRNA expression analysis was performed using standard qRT-PCR procedures, as described earlier [6], using specific primer sets, with U6 serving as the housekeeping gene for miRNA normalization. A 5 µL volume of cDNA was used as a template for PCR without dilution, using the CFX96 Touch Real-Time PCR Detection System (Bio-Rad, Hercules, CA, USA) in a total reaction volume of 20 µL, which included 10 µL of SYBR Green qPCR Master Mix (2×) and specific forward and reverse primer sets listed in Table 1. The thermal cycling conditions were as follows: cycle 1 at 95°C for 10 min, followed by 40 cycles at 95°C for 10 s and 56°C/60°C for 45 s, followed by melting curve analysis. The fluorescence signal was represented in the form of a cycle threshold (Ct) value.

Details of the tumour marker assays are given in Table 2.

**Table 2:** Details of the various tumour marker assays used. CA 19-9: Carbohydrate antigen 19-9; CEA: Carcinoembryonic antigen; CA-125: Cancer antigen 125; CMIA: Chemiluminescent microparticle immunoassay.

Parameter	CA 19-9	CEA	CA-125
Manufacturer/assay	Abbott ARCHITECT CA 19-9XR	Abbott ARCHITECT CEA	Abbott ARCHITECT CA 125 II
Kit/catalogue number	02K91-32	07K68-27	2K45-29
Detection range	2.00-1,200.00 U/mL	0.50-1,500.00 ng/mL	1.0-1,000 U/mL
Sample type	Serum	Serum	Serum
Analytical method	CMIA	CMIA	CMIA
Calibration	Calibrator set (02K9103)	Calibrator set (07K6802)	Calibrator set (02K4502)
Quality control procedures	Testing a single sample at each control level once every 24 hours on each day of use	Running multilevel control materials, evaluating results against established statistical parameters, and performing routine instrument maintenance to ensure precise CMIA results	Testing a single sample at each control level once every 24 hours on each day of use

All collected data were systematically compiled for analysis.

Control group

Biomarker analysis was performed using the same protocol as that used for cases to maintain uniformity.

Outcome measures

The primary outcome was the identification of non-invasive biomarkers useful for the early diagnosis of GBC. Secondary outcomes included comparison of biomarker levels between malignant and benign conditions and evaluation of diagnostic accuracy, including sensitivity, specificity, and predictive values.

Pilot study

A pilot study was conducted on a small subset of patients to assess feasibility, refine methodology, and standardize data collection tools. Necessary modifications were made before initiating the main study.

Validity and reliability

Content validity was ensured through expert review of the study protocol and data collection tools. Standardized laboratory procedures and calibrated equipment were used to ensure accuracy. All biomarker analyses were performed using validated techniques, such as ELISA and qRT-PCR. Repeated measurements and internal quality control were maintained to ensure reliability and minimize errors.

Statistical analysis

Data were entered into Microsoft Excel and analysed using SPSS version 24. Continuous variables were expressed as mean ± SD and compared using Student’s t-test or the Mann-Whitney U test. Categorical variables were expressed as percentages and analysed using the chi-square test or Fisher’s exact test. Diagnostic accuracy was assessed using ROC curve analysis. Correlations between biomarker levels and tumour stage or histological grade were analysed using Pearson or Spearman correlation. A p-value of <0.05 was considered statistically significant.

## Results

The baseline demographic characteristics of the two groups and their comparison are shown in Table 3.

**Table 3:** Baseline demographic characteristics (age-matched groups). Statistical tests used: Independent-samples t-test for age and chi-square (χ²) test for categorical variables. A p-value of less than 0.05 was considered statistically significant. GBC: Gallbladder carcinoma.

Characteristic	GBC group (n = 30)	Control group (n = 30)	p-value	Test statistic
Age (years), mean ± SD	56.4 ± 9.8	54.2 ± 10.6	0.403	t = 0.8347
Age >50 years, n (%)	22 (73.3%)	19 (63.3%)	0.405	χ² = 0.693
Female sex, n (%)	24 (80.0%)	23 (76.7%)	0.754	χ² = 0.098
Rural residence, n (%)	19 (63.3%)	16 (53.3%)	0.432	χ² = 0.617
Socioeconomic status (Modified Kuppuswamy), n (%)				
Lower class (I-II)	6 (20.0%)	7 (23.3%)	0.754	χ² = 0.098
Middle class (III-IV)	18 (60.0%)	19 (63.3%)	0.791	χ² = 0.071
Upper class (V)	6 (20.0%)	4 (13.3%)	0.488	χ² = 0.480
Occupation, n (%)				
Homemaker	18 (60.0%)	16 (53.3%)	0.602	χ² = 0.271
Agricultural worker	7 (23.3%)	6 (20.0%)	0.754	χ² = 0.098
Manual labourer	3 (10.0%)	5 (16.7%)	0.448	χ² = 0.577
Business/professional	2 (6.7%)	3 (10.0%)	0.64	χ² = 0.218

In the GBC group, the mean age was 56.4 ± 9.8 years compared to 54.2 ± 10.6 years in the control group (p = 0.403). A total of 22 (73.3%) cases were above the age of 50 years compared to 19 (63.3%) controls (p = 0.405). In both groups, females predominated, with 24 (80.0%) in the GBC group and 23 (76.7%) in the control group (p = 0.754). A total of 19 (63.3%) cases and 16 (53.3%) controls (p = 0.432) belonged to rural areas. The majority of patients in both groups belonged to the middle class (60% in the GBC group vs. 63.3% in the control group, p = 0.791). The majority were female homemakers (60% in the GBC group vs. 53.3% in the control group, p = 0.602).

The duration of presenting symptoms is shown in Table 4. Patients in the GBC group had a significantly longer duration of symptoms than those in the control group.

**Table 4:** Duration of presenting symptoms. Statistical test used: Mann-Whitney U test (for non-parametric distribution). A p-value of less than 0.05 was considered statistically significant. GBC: Gallbladder carcinoma.

Symptom duration (weeks)	GBC group (n = 30), mean ± SD	Control group (n = 30), mean ± SD	p-value	U statistic
Abdominal pain	8.4 ± 4.2	6.1 ± 3.5	0.0004	U = 142.50
Jaundice	3.2 ± 1.8	1.4 ± 1.1	<0.001	U = 11.00
Weight loss	5.7 ± 2.9	2.3 ± 1.6	<0.001	U = 10.50
Loss of appetite	6.1 ± 3.1	1.9 ± 1.4	<0.001	U = 0.00
Fever	2.8 ± 1.5	1.6 ± 0.9	<0.001	U = 110

The clinical symptoms of the cases and controls are shown in Table 5. Symptoms such as jaundice, loss of weight and appetite, pruritus, and dark-coloured urine or pale stools were more common in GBC patients than in controls.

**Table 5:** Clinical presentation profile. Statistical test used: Chi-square (χ²) test. A p-value of less than 0.05 was considered statistically significant. GBC: Gallbladder carcinoma.

Symptom (present)	GBC group (n = 30)	Control group (n = 30)	p-value	Test statistic
Abdominal pain	28 (93.3%)	29 (96.7%)	0.554	χ² = 0.351
Jaundice	21 (70.0%)	6 (20.0%)	<0.001	χ² = 15.152
Weight loss	25 (83.3%)	8 (26.7%)	<0.001	χ² = 19.461
Loss of appetite	27 (90.0%)	12 (40.0%)	<0.001	χ² = 16.484
Fever	14 (46.7%)	9 (30.0%)	0.184	χ² = 1.763
Nausea/vomiting	16 (53.3%)	15 (50.0%)	0.796	χ² = 0.067
Pruritus	12 (40.0%)	3 (10.0%)	0.007	χ² = 7.200
Dark urine/pale stools	15 (50.0%)	4 (13.3%)	0.002	χ² = 9.320

The comorbid conditions and family history are shown in Table 6. A history of GBC in a first-degree relative was significantly more common among cases (p < 0.05). None of the controls had a positive family history of GBC.

**Table 6:** Comorbidities and family history. Statistical test used: Chi-square (χ²) test. A p-value of less than 0.05 was considered statistically significant.

Parameter	GBC group (n = 30)	Control group (n = 30)	p-value	Test statistic
Comorbidities present				
Diabetes mellitus	10 (33.3%)	8 (26.7%)	0.573	χ² = 0.317
Hypertension	9 (30.0%)	7 (23.3%)	0.559	χ² = 0.341
Hypothyroidism	4 (13.3%)	3 (10.0%)	0.688	χ² = 0.162
Family history of cancer	7 (23.3%)	2 (6.7%)	0.071	χ² = 3.268
- Gallbladder cancer in a first-degree relative	4 (13.3%)	0 (0%)	0.038	χ² = 4.286
- Other gastrointestinal malignancy	3 (10.0%)	2 (6.7%)	0.641	χ² = 0.218
Past surgical history	12 (40.0%)	8 (26.7%)	0.273	χ² = 1.200
- Previous cholecystectomy	2 (6.7%)	0 (0%)	0.15	χ² = 2.069
- Other abdominal surgery	10 (33.3%)	8 (26.7%)	0.573	χ² = 0.317

The findings on clinical examination, including ECOG status, are shown in Table 7. The findings demonstrate that GBC patients were more frequently icteric and had a palpable abdominal lump. The majority of patients in the control group were fully active (ECOG 0) (p < 0.001), while in the GBC group, the majority had an ECOG score of 2 (40%).

**Table 7:** General physical examination and ECOG performance status. Statistical test used: Chi-square (χ²) test for trend for ECOG performance status scores. A p-value of less than 0.05 was considered statistically significant. ECOG: Eastern Cooperative Oncology Group.

Examination finding (present)	Cases (n = 30)	Controls (n = 30)	p-value	Test statistic
Icterus	22 (73.3%)	5 (16.7%)	<0.001	χ² = 19.461
Abdominal tenderness	24 (80.0%)	27 (90.0%)	0.278	χ² = 1.176
Palpable lump	17 (56.7%)	2 (6.7%)	<0.001	χ² = 17.330
- Right hypochondrial lump	14 (46.7%)	2 (6.7%)	<0.001	χ² = 12.273
- Epigastric lump	3 (10.0%)	0 (0%)	0.0756	χ² = 3.158
ECOG performance status				
ECOG 0 (Fully active)	2 (6.7%)	18 (60.0%)	<0.001	χ² = 19.200
ECOG 1 (Ambulatory, light work)	8 (26.7%)	10 (33.3%)	0.573	χ² = 0.317
ECOG 2 (Ambulatory, self-care)	12 (40.0%)	2 (6.7%)	0.002	χ² = 9.317
ECOG 3 (Limited self-care)	6 (20.0%)	0 (0%)	0.009	χ² = 6.667
ECOG 4 (Completely disabled)	2 (6.7%)	0 (0%)	0.15	χ² = 2.069

The haematological parameters and liver chemistry findings are shown in Table 8. All haematological parameters and liver biochemical parameters were significantly more deranged in the GBC group than in controls.

**Table 8:** Haematological tests and liver function tests. Statistical tests used: Independent-samples t-test (parametric) and Mann-Whitney U test (nonparametric) for neutrophil-to-lymphocyte ratio (NLR), platelet-to-lymphocyte ratio (PLR), bilirubin, and alkaline phosphatase (ALP). A p-value of less than 0.05 was considered statistically significant. GBC: Gallbladder carcinoma; NLR: Neutrophil-to-lymphocyte ratio; PLR: Platelet-to-lymphocyte ratio; AST: Aspartate aminotransferase; ALT: Alanine aminotransferase; SGOT: Serum glutamic-oxaloacetic transaminase; SGPT: Serum glutamic-pyruvic transaminase; ALP: Alkaline phosphatase.

Laboratory parameter	GBC group, mean ± SD	Control group, mean ± SD	Normal range	p-value	Test statistic
Haemoglobin (g/dL)	10.2 ± 1.8	12.4 ± 1.2	12.0-16.0	<0.001	t = 5.570
Total leucocyte count (/mm³)	9,842 ± 3,214	7,856 ± 2,103	4,000-11,000	0.006	t = 2.832
Platelet count (lakhs/mm³)	3.2 ± 1.1	2.4 ± 0.8	1.5-4.5	0.002	t = 3.221
Neutrophil-to-lymphocyte ratio (NLR)	3.8 ± 1.4	2.1 ± 0.7	<2.0	<0.001	U = 22.00
Platelet-to-lymphocyte ratio (PLR)	178.4 ± 52.3	124.6 ± 38.1	<150	<0.001	U = 78.00
Total bilirubin (mg/dL)	3.8 ± 2.9	0.9 ± 0.4	0.2-1.2	<0.001	U = 51.50
Direct bilirubin (mg/dL)	2.6 ± 2.1	0.3 ± 0.2	0.0-0.3	<0.001	U = 4.50
Aspartate aminotransferase (AST/SGOT) (IU/L)	84.6 ± 42.3	32.4 ± 12.7	Up to 40	<0.001	t = 6.473
Alanine aminotransferase (ALT/SGPT) (IU/L)	72.4 ± 38.6	34.2 ± 14.5	Up to 40	<0.001	t = 5.074
Alkaline phosphatase (ALP) (IU/L)	214.6 ± 78.4	98.3 ± 28.6	45-120	<0.001	U = 37.50
Serum albumin (g/dL)	2.9 ± 0.6	4.1 ± 0.4	3.5-5.0	<0.001	t = 9.115
AST/ALT ratio	1.22 ± 0.35	0.97 ± 0.28	<1.0	0.004	t = 3.055

The tumour marker levels are shown in Table 9. All conventional tumour markers were significantly elevated in the GBC group (p < 0.001). The ΔCt (delta cycle threshold) values of non-invasive biomarkers, such as miRNA-21 and miRNA-155, were significantly lower in cases than in controls (p < 0.001). A lower ΔCt value is suggestive of higher miRNA expression in cases, and vice versa in controls.

**Table 9:** Tumour biomarker levels. Statistical test used: Mann-Whitney U test, as all biomarkers exhibited non-normal distributions. A p-value of less than 0.05 was considered statistically significant. GBC: Gallbladder carcinoma; CA 19-9: Carbohydrate antigen 19-9; CEA: Carcinoembryonic antigen; CA-125: Cancer antigen 125; miRNA: MicroRNA; ΔCt: Difference in cycle threshold; N/A: Not applicable.

Biomarker	GBC group, mean ± SD	Control group, mean ± SD	Normal range	p-value	Test statistic
CA 19-9 (U/mL)	214.6 ± 142.8	22.4 ± 12.6	<37 U/mL	<0.001	U = 0.00
CEA (ng/mL)	8.6 ± 5.2	2.1 ± 1.1	<5 ng/mL	<0.001	U = 5.50
CA-125 (U/mL)	58.4 ± 32.6	18.6 ± 10.2	<35 U/mL	<0.001	U = 9.50
miRNA-21 (ΔCt)	0.98 ± 0.42	3.82 ± 1.24	N/A	<0.001	U = 0.00
miRNA-155 (ΔCt)	0.76 ± 0.34	2.94 ± 1.08	N/A	<0.001	U = 0.00

The USG findings of both groups are shown in Table 10. Of the 30 cases, 28 (93.3%) had a thickened gallbladder (GB) wall (>4 mm). A total of 60% of cases had coexisting gallstone disease. Porcelain GB was present in only 20% of cases. One-third of cases had suspected liver metastasis, and more than 50% had dilated intrahepatic biliary radicles (16 out of 30 cases).

**Table 10:** Imaging findings on USG. Statistical test used: Chi-square (χ²) test. A p-value of less than 0.05 was considered statistically significant. GBC: Gallbladder carcinoma; GB: Gallbladder; IHBRD: Intrahepatic biliary radicle dilatation.

USG finding	GBC group, n (%)	Control group, n (%)	p-value	Test statistic
GB wall thickness > 4 mm	28 (93.3%)	12 (40.0%)	<0.001	χ² = 19.200
Intraluminal mass/polyp	22 (73.3%)	1 (3.3%)	<0.001	χ² = 31.093
GB calculi present	18 (60.0%)	30 (100%)	<0.001	χ² = 15.000
Porcelain GB	6 (20.0%)	1 (3.3%)	0.044	χ² = 4.043
Pericholecystic collection	8 (26.7%)	2 (6.7%)	0.038	χ² = 4.320
Suspected liver metastasis	10 (33.3%)	0 (0%)	<0.001	χ² = 12.000
Biliary dilatation (IHBRD)	16 (53.3%)	3 (10.0%)	<0.001	χ² = 13.017

Computed tomography (CT) findings of both groups are shown in Table 11. All cases had several features on contrast-enhanced computed tomography (CECT) suggestive of malignancy, which were absent in the control group.

**Table 11:** Contrast-enhanced computed tomography (CECT) findings of the abdomen. Statistical test used: Chi-square (χ²) test. A p-value of less than 0.05 was considered statistically significant. CECT: Contrast-enhanced computed tomography; GBC: Gallbladder carcinoma.

CECT finding of the abdomen	GBC group (n = 30), n (%)	Control group (n = 30), n (%)	p-value	Test statistic
Enhancing gallbladder mass	28 (93.3%)	0 (0%)	<0.001	χ² = 52.5
Liver infiltration	27 (90%)	0 (0%)	<0.001	χ² = 49.091
Nodal involvement	23 (76.6%)	0 (0%)	<0.001	χ² = 37.29
- Porta hepatis lymph nodes	16 (53.3%)	0 (0%)	<0.001	χ² = 21.812
- Retroperitoneal lymph nodes	10 (33.3%)	0 (0%)	0.003	χ² = 12.0
Vascular encasement (portal vein/hepatic artery)	9 (30%)	0 (0%)	0.001	χ² = 10.589
Distant metastasis	10 (33.3%)	0 (0%)	0.003	χ² = 12.0

Table 12 shows the TNM staging of the cases. The majority of patients had T4 disease (33.3%) at the time of diagnosis. N1 disease was present in 36.7% of patients, followed by N2 (33.3%) and N0 (30%) stages. The majority of patients did not have distant metastasis at the time of diagnosis (66.7%). A total of 70% of patients had either stage III or IV disease at the time of presentation.

**Table 12:** TNM staging distribution (GBC group only; AJCC 8th edition). Statistical test used: Descriptive statistics only (single group). TNM: Tumour-node-metastasis; GBC: Gallbladder carcinoma; AJCC: American Joint Committee on Cancer.

TNM parameter	Category	Number of patients (n = 30)	Percentage (%)
Tumour (T) stage	T1a/T1b	5	16.70%
	T2a/T2b	8	26.70%
	T3	7	23.30%
	T4	10	33.30%
Node (N) stage	N0	9	30%
	N1	11	36.70%
	N2	10	33.30%
Metastasis (M) stage	M0	20	66.70%
	M1	10	33.30%
Overall stage	Stage I	5	16.70%
	Stage II	4	13.30%
	Stage IIIA	0	0%
	Stage IIIB	11	36.70%
	Stage IVA/IVB	10	33.30%

The tumour type is shown in Figure 1, and its grade is shown in Figure 2. The other histological characteristics are shown in Table 13. Well-differentiated tumours were present in 6 (20.0%) cases, moderately differentiated tumours in 13 (43.3%) cases, and poorly differentiated tumours in 11 (36.7%) cases. Histological types included adenocarcinoma (NOS) in 24 (80.0%) cases, papillary adenocarcinoma in 3 (10.0%) cases, mucinous adenocarcinoma in 2 (6.7%) cases, and squamous/adenosquamous carcinoma in 1 (3.3%) case. Lymphovascular invasion was present in 16 (53.3%) cases, and perineural invasion in 14 (46.7%) cases. Resection margin status among surgical patients showed R0 (negative) in 8 (88.9%) and R1/R2 (positive) in 1 (11.1%).

**Figure 1:**
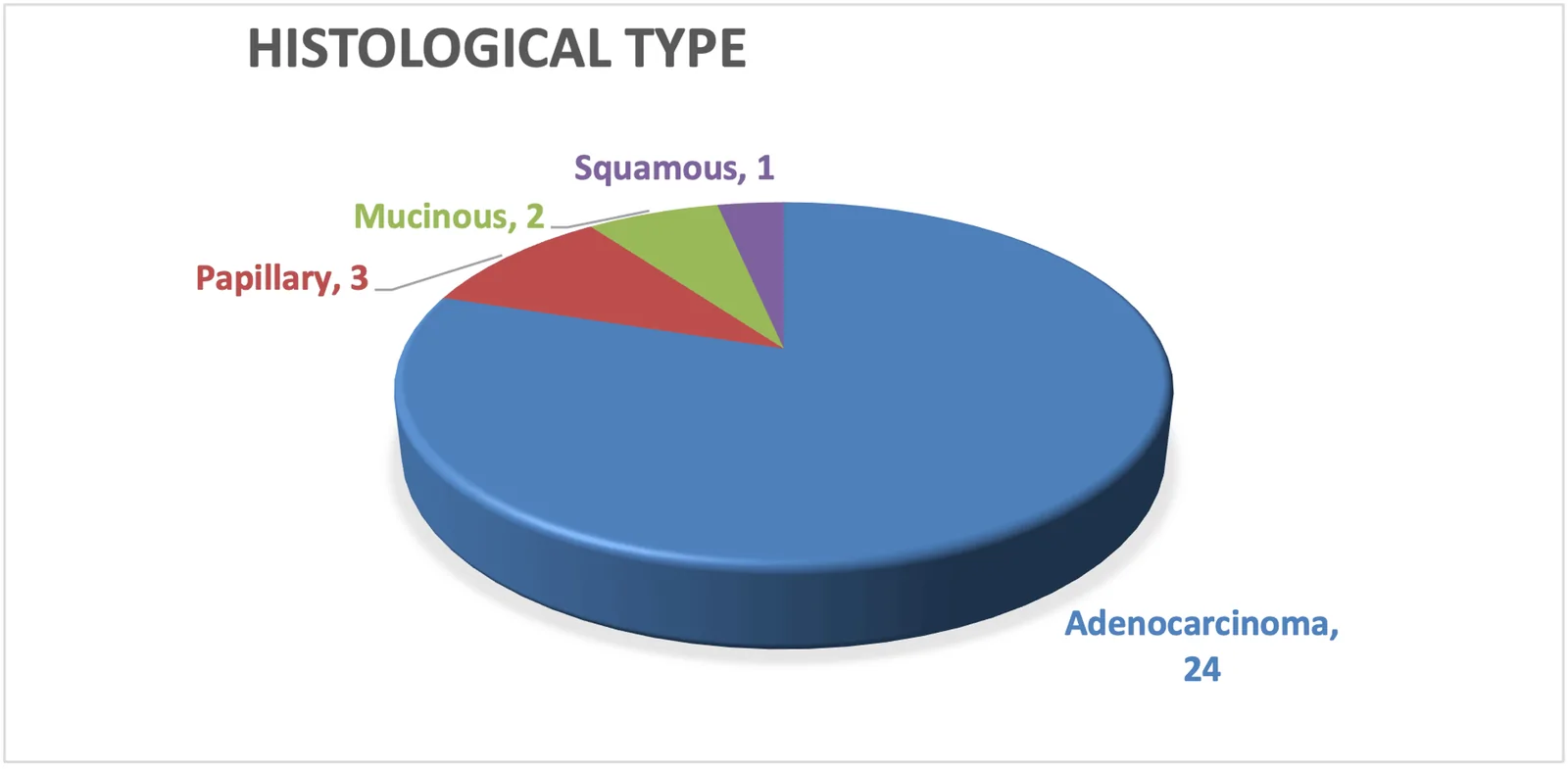
Tumour characteristics (GBC group). GBC: Gallbladder carcinoma.

**Figure 2:**
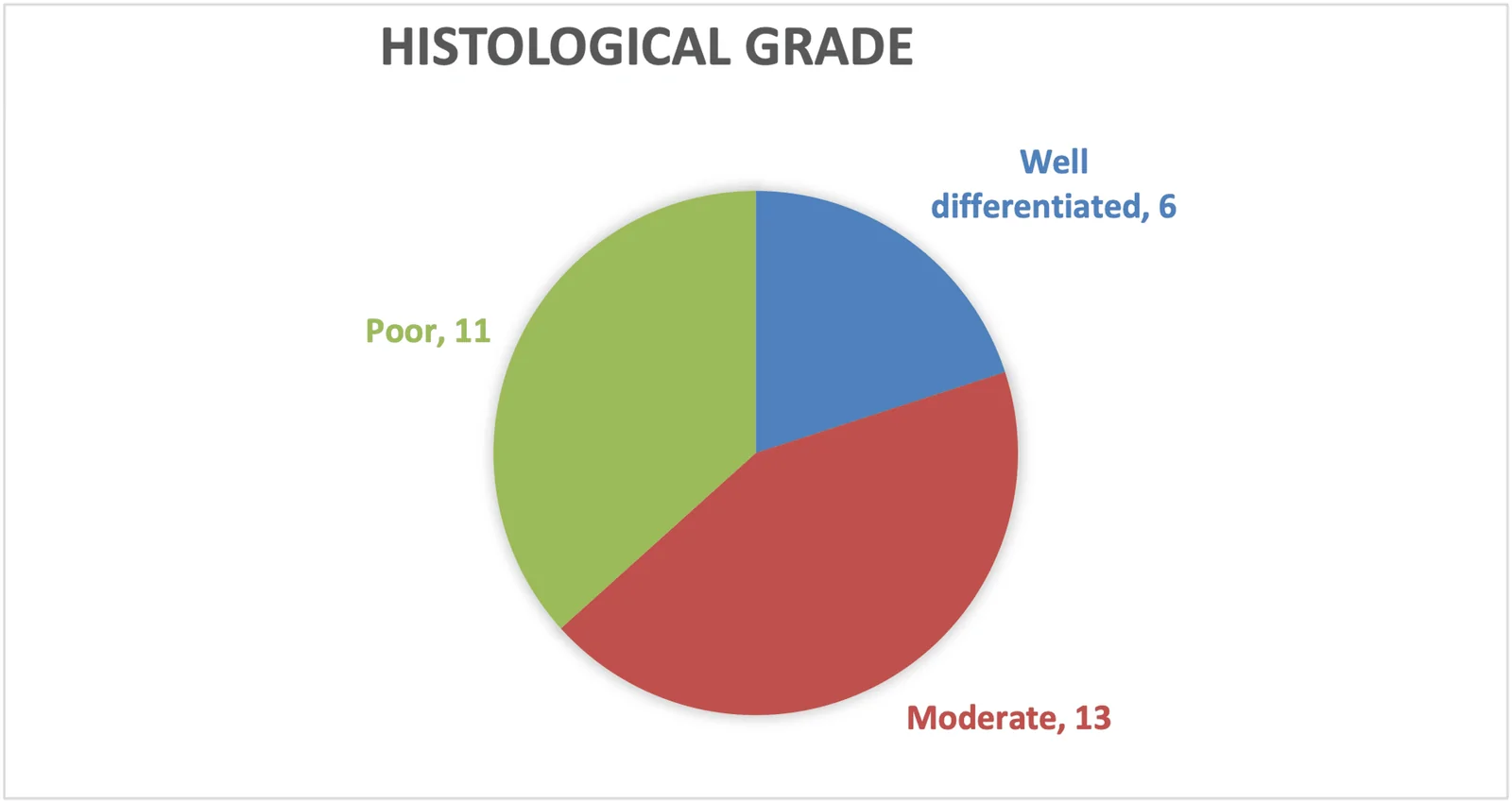
Histological grade (GBC group). GBC: Gallbladder carcinoma.

**Table 13:** Tumour characteristics (GBC group). Statistical test used: Descriptive statistics. GBC: Gallbladder carcinoma.

Histopathological parameter	Subtype	Number (n = 30)	Percentage (%)
Lymphovascular invasion	Present	16	53.30%
Perineural invasion	Present	14	46.70%
Resection margin status (surgical patients, n = 9)	R0 (negative)	8	88.90%
	R1/R2 (positive)	1	11.10%

The levels of tumour markers according to tumour stage are shown in Figure 3. There was a significant increase in the levels of various tumour markers with advancing disease stage. The ΔCt values of miRNA-21 progressively decreased (indicating increased expression) as the disease stage advanced.

**Figure 3:**
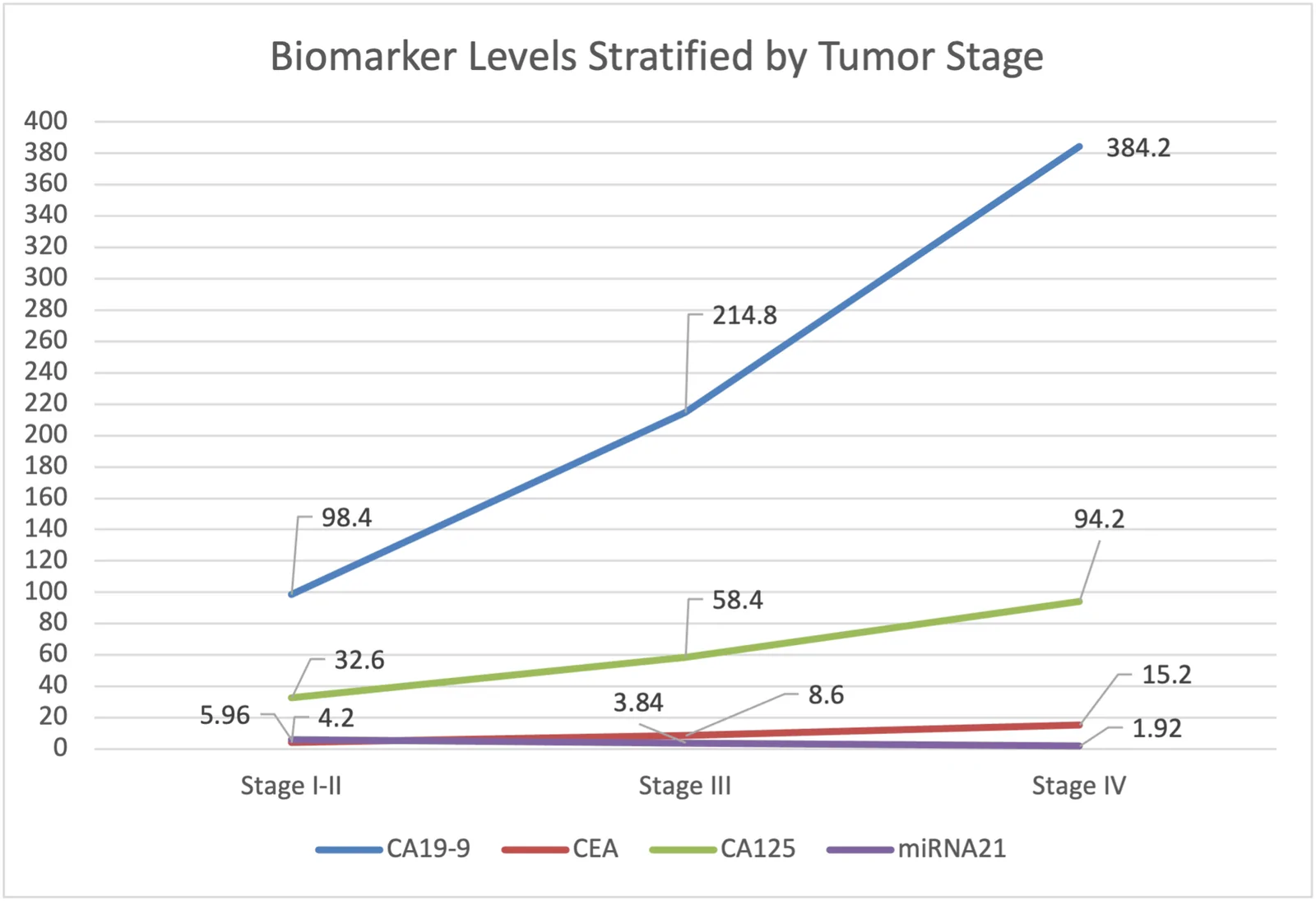
Biomarker levels stratified by tumour stage (GBC group only). Statistical tests used: Kruskal-Wallis test followed by Dunn’s post hoc test. The reported p-value for trend was less than 0.001 for all biomarkers. GBC: Gallbladder carcinoma.

Table 14 compares the diagnostic performance of individual tumour markers and their combinations. Among all study participants, CA 19-9 showed an AUC of 0.892 with a cutoff of >42.5 U/mL, sensitivity of 86.7%, specificity of 83.3%, and accuracy of 85.0% (p < 0.001). CEA showed an AUC of 0.784 with a cutoff of >4.2 ng/mL, sensitivity of 73.3%, specificity of 76.7%, and accuracy of 75.0% (p < 0.001). CA-125 showed an AUC of 0.826 with a cutoff of >32.0 U/mL, sensitivity of 76.7%, specificity of 80.0%, and accuracy of 78.3% (p < 0.001). miRNA-21 showed an AUC of 0.914 with a ΔCt cutoff value of <1.85. At this cutoff, the sensitivity was 90.0%, specificity was 86.7%, and accuracy was 88.3% (p < 0.001). The combined panel (CA 19-9 + miRNA-21) showed an AUC of 0.956 with sensitivity of 93.3%, specificity of 90.0%, and accuracy of 91.7% (p < 0.001).

**Table 14:** Diagnostic performance of individual biomarkers (ROC curve analysis). Statistical analysis: Nonparametric ROC curve analysis was performed. DeLong’s method was used to compare the AUCs, and the Youden J index was used to determine the optimal cut-off values. A binary logistic regression model was used to evaluate the combined diagnostic performance of CA 19-9 and miR-21. A p-value < 0.05 was considered statistically significant. AUC: Area under the curve; CA 19-9: Carbohydrate antigen 19-9; CA 125: Cancer antigen 125; CEA: Carcinoembryonic antigen; ΔCt: Delta cycle threshold; LR+: Positive likelihood ratio; LR-: Negative likelihood ratio; miRNA-21: MicroRNA-21; NPV: Negative predictive value; PPV: Positive predictive value; ROC: Receiver operating characteristic.

Biomarker	AUC (95% CI)	Cut-off value	Sensitivity (%)	Specificity (%)	LR+	LR−	PPV (%)	NPV (%)	Accuracy (%)	p-value
CA 19-9	0.892 (0.808-0.976)	>42.5 U/mL	86.7	83.3	5.19	0.16	83.9	86.2	85	<0.001
CEA	0.784 (0.678-0.890)	>4.2 ng/mL	73.3	76.7	3.15	0.35	75.9	74.2	75	<0.001
CA 125	0.826 (0.724-0.928)	>32.0 U/mL	76.7	80	3.84	0.29	79.3	77.4	78.3	<0.001
miRNA-21	0.914 (0.842-0.986)	<1.85 ΔCt	90	86.7	6.77	0.12	87.1	89.7	88.3	<0.001
Combined panel (CA 19-9 + miRNA-21)	0.956 (0.904-1.000)	Logit >0.62	93.3	90	9.33	0.07	90.3	93.1	91.7	<0.001

Table 15 compares various markers between surgical and nonsurgical subgroups among the cases. The levels of tumour markers were significantly higher in the unresectable group. The ΔCt value of miRNA-21 was significantly lower in unresectable cases (indicating higher expression of miRNA-21), consistent with a more advanced stage of disease.

**Table 15:** Comparison of biomarker levels between resectable and unresectable disease (GBC group). Resectability is defined as R0 resection possible on staging; unresectable includes locally advanced (T4/N2/M1) disease. Statistical analysis: The Mann-Whitney U test. A p-value < 0.05 was considered statistically significant. CA 19-9: Carbohydrate antigen 19-9; CA 125: Cancer antigen 125; CEA: Carcinoembryonic antigen; ΔCt: Delta cycle threshold; GBC: Gallbladder carcinoma; miRNA-21: MicroRNA-21; U: Mann-Whitney U statistic.

Biomarker	Resectable (n = 9), mean ± SD	Unresectable (n = 21), mean ± SD	p-value	Test statistic
CA 19-9 (U/mL)	98.4 ± 42.6	264.4 ± 138.2	<0.001	U = 4.00
CEA (ng/mL)	4.2 ± 1.8	10.5 ± 5.4	<0.001	U = 3.50
CA 125 (U/mL)	32.6 ± 12.4	69.6 ± 31.8	0.002	U = 3.00
miRNA-21 (ΔCt)	4.53 ± 1.42	1.92 ± 0.68	<0.001	U = 4.00

The different treatment modalities (surgical and nonsurgical) are shown in Table 16. Curative-intent surgery was performed in only nine patients (30%) in the GBC group, while 28 patients (93.3%) in the control group underwent laparoscopic cholecystectomy. Two patients (6.7%) in the control group did not undergo surgery due to logistical reasons and were provided with best supportive care. Fourteen patients (46.7%) in the GBC group had advanced disease and received palliative combination chemotherapy consisting of gemcitabine and cisplatin. Seven patients (23.3%) in the GBC group received best supportive care. Of the nine patients in the surgical group, one underwent laparoscopic cholecystectomy, two underwent open cholecystectomy, and six underwent radical cholecystectomy (standard lymphadenectomy with a 2 cm liver wedge resection).

**Table 16:** Treatment modalities and planned interventions Statistical analysis: The chi-square (χ²) test. A p-value < 0.05 was considered statistically significant. GBC: Gallbladder carcinoma.

Treatment parameter	GBC group (n = 30), n (%)	Control group (n = 30), n (%)	p-value	Test statistic
Primary treatment planned				
Curative-intent surgery	9 (30.0%)	28 (93.3%)	<0.001	χ² = 25.452
Palliative chemotherapy (gemcitabine + cisplatin)	14 (46.7%)	0 (0%)	<0.001	χ² = 18.261
Best supportive care	7 (23.3%)	2 (6.7%)	0.071	χ² = 3.268
Type of surgery performed (surgical patients only)				
Laparoscopic cholecystectomy	1 (of 9)	28 (of 28)	<0.001	χ² = 31.755
Open cholecystectomy	2 (of 9)	0 (of 28)	0.01	χ² = 6.578
Radical cholecystectomy with lymphadenectomy	6 (of 9)	0 (of 28)	<0.001	χ² = 22.280

The treatment response and follow-up outcomes of the cases (n = 30) are shown in Table 17. Eight patients showed a partial or complete response (26.7%). The status at the last follow-up showed that 12 patients (40.0%) were alive. Fourteen patients (46.7%) had died due to the disease. The median survival was 8.4 ± 2.2 months among the cases.

**Table 17:** Treatment response and follow-up outcomes Statistical analysis: Kaplan-Meier survival analysis was performed to estimate median survival, and the log-rank test was used to compare survival distributions between groups. A p-value < 0.05 was considered statistically significant. GBC: Gallbladder carcinoma.

Follow-up parameter (at 6 months)	GBC group (n = 30), n (%)
Treatment response (treated patients)	
Responder (partial/complete response)	8 (26.7%)
Stable disease	6 (20.0%)
Progressive disease	11 (36.7%)
Not evaluable (lost to follow-up)	5 (16.7%)
Status at last follow-up	
Alive	12 (40.0%)
Deceased (disease-related)	14 (46.7%)
Deceased (other causes)	1 (3.3%)
Lost to follow-up	3 (10.0%)
Median survival (months)	8.4 (95% CI: 6.2-10.6)

The correlation of various markers with tumour grade is shown in Table 18. All tumour markers showed a significant positive correlation with tumour grade. Serum albumin and miRNA-21 showed a significant negative correlation with histological grade (p < 0.001).

**Table 18:** Correlation between preoperative biomarker levels and postoperative histological grade. Statistical analysis: Spearman’s rank correlation coefficient was used to assess the association between preoperative biomarker levels and postoperative histological grade. A p-value < 0.05 was considered statistically significant. CA 19-9: Carbohydrate antigen 19-9; CA 125: Cancer antigen 125; CEA: Carcinoembryonic antigen; ΔCt: Delta cycle threshold; miRNA-21: MicroRNA-21.

Preoperative biomarker	Correlation with histological grade (1 = well differentiated, 2 = moderately differentiated, 3 = poorly differentiated)	Correlation Coefficient (r)	95% CI	p-value
CA 19-9 (U/mL)	Positive correlation with higher grade	0.642	0.334 to 0.826	<0.001
CEA (ng/mL)	Positive correlation with higher grade	0.534	0.190 to 0.761	0.002
CA 125 (U/mL)	Positive correlation with higher grade	0.486	0.131 to 0.730	0.006
miRNA-21 (ΔCt)	Negative correlation with higher grade	-0.718	-0.868 to -0.447	<0.001
Total bilirubin (mg/dL)	Weak positive correlation	0.312	-0.063 to 0.610	0.094
Albumin (g/dL)	Negative correlation (lower albumin levels associated with higher grade)	-0.594	-0.798 to -0.268	<0.001

The levels of tumour markers were compared between carcinoma patients with and without gallstones, as shown in Table 19. There was no significant difference in tumour marker levels or ΔCt values of miRNA-21 between GBC patients with and without gallstones.

**Table 19:** Biomarker levels in patients with and without gallstones (GBC group). Statistical analysis: An independent-samples t-test was used to compare biomarker levels between patients with and without gallstones. A p-value < 0.05 was considered statistically significant. CA 19-9: Carbohydrate antigen 19-9; CA 125: Cancer antigen 125; CEA: Carcinoembryonic antigen; ΔCt: Delta cycle threshold; GBC: Gallbladder carcinoma; miRNA-21: MicroRNA-21.

Biomarker	GBC with gallstones (n = 18), mean ± SD	GBC without gallstones (n = 12), mean ± SD	p-value	Test statistic
CA 19-9 (U/mL)	198.4 ± 128.6	238.6 ± 162.4	0.456	t = 0.755
CEA (ng/mL)	7.8 ± 4.6	9.8 ± 6.2	0.319	t = 1.015
CA 125 (U/mL)	54.2 ± 29.4	64.8 ± 36.8	0.389	t = 0.875
miRNA-21 (ΔCt)	4.12 ± 1.36	3.62 ± 1.18	0.293	t = 1.070

## Discussion

Non-invasive biomarkers are molecules or signatures that can be measured in accessible body fluids and reflect the presence, biological behaviour, or burden of neoplasia. In GBC, the multistep progression from chronic inflammation to dysplasia to carcinoma creates molecular alterations (genetic, epigenetic, and proteomic) that are candidate targets for biomarker discovery [7]. Examples include classical serum tumour antigens, inflammatory cytokines, circulating cell-free DNA (cfDNA) or circulating tumour DNA (ctDNA), circulating microRNAs (miRNAs), exosomal contents, and proteomic or metabolomic signatures detectable using modern platforms (Figure 4) [8]. Non-invasive biomarkers should ideally be reproducible, cost-effective, and translatable to routine clinical laboratories in regions where the GBC burden is high [9].

**Figure 4:**
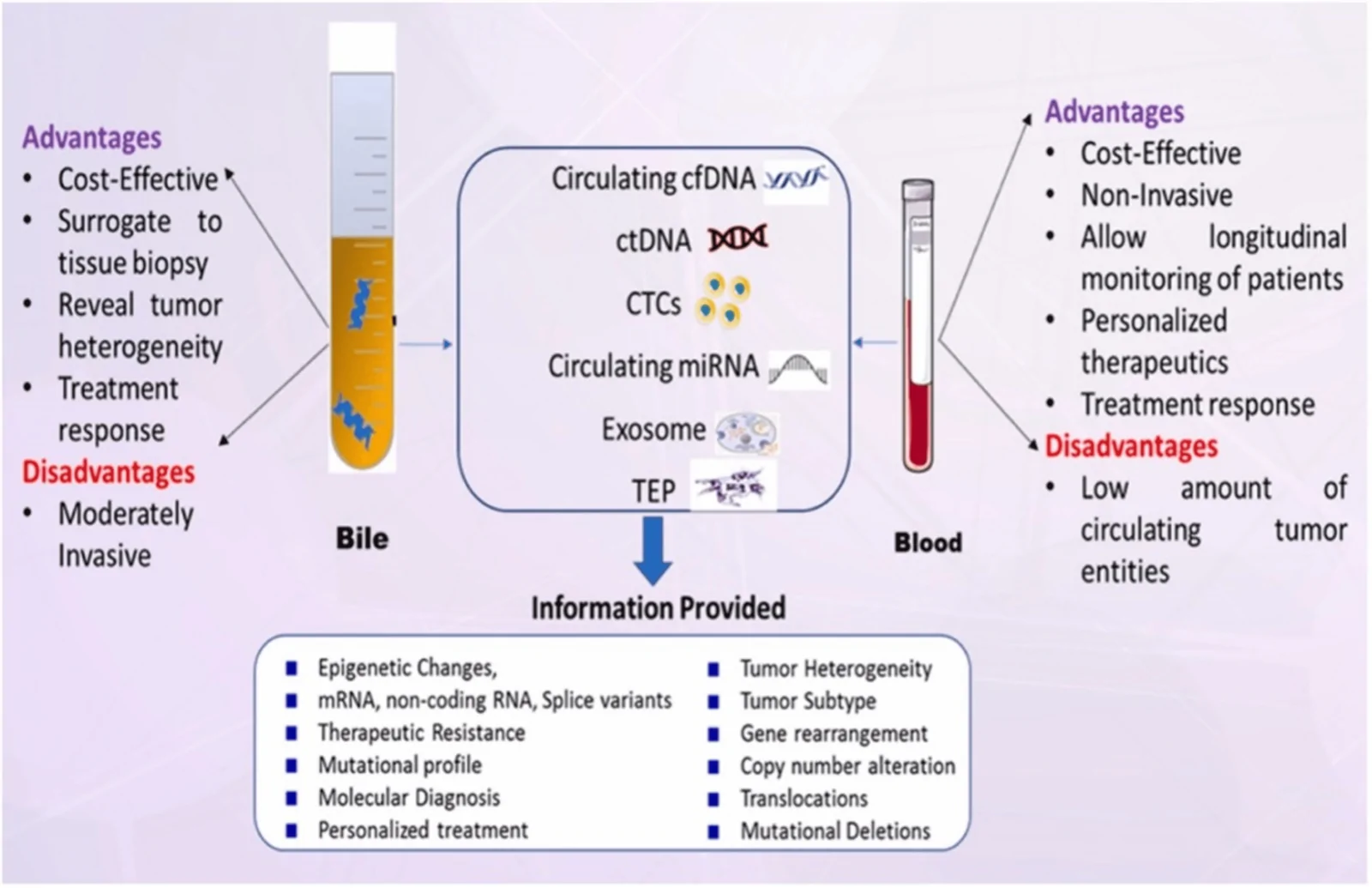
Liquid biopsy component. Source: Reference [8]. ctDNA: Circulating tumour DNA; CTCs: Circulating tumour cells; TEP: Tumour-educated platelets; cfDNA: Cell-free DNA; mRNA: Messenger RNA.

In the present study, carcinoma patients had a higher mean age of 56.4 ± 9.8 years compared to 54.2 ± 10.6 years in controls, with a predominance of females (80% vs. 76.7%). Rawla P et al. [10] reported a comparable age range (55-60 years) and gender distribution (75% females). Rural predominance was also observed (63.3% vs. 53.3%). This was higher than the 55% reported in Indian cohorts by Kumar [1], suggesting possible environmental or socioeconomic influences. Socioeconomic distribution in our study showed that 60% belonged to the middle class, similar to the 58.2% reported by Sinha SR et al. [11], while lower-class representation of 20% was comparable to the 18.5% observed by Bind MK et al. [12]. The proportion of homemakers (60%) was slightly higher than the 52% reported in Indian epidemiological data by Kumar A et al. [1]. Overall, our findings align with the existing literature, confirming that GBC predominantly affects older females from rural and middle socioeconomic backgrounds, although our study showed slightly higher female and rural representation, possibly due to regional variation.

Clinically, GBC patients presented with significantly longer symptom durations, including abdominal pain (8.4 ± 4.2 weeks), jaundice (3.2 ± 1.8 weeks), and weight loss (5.7 ± 2.9 weeks). These durations were longer than those reported in other studies [1, 2, 11-13]. These differences suggest that patients in our study presented at a relatively later stage. Studies by Rawla P et al. [10] and Kumar A et al. [1] have emphasised that delayed healthcare access and a lack of early screening contribute to prolonged symptom duration in GBC. Our findings are consistent with these observations, reinforcing that longer symptom duration is a key clinical indicator of malignancy.

The prevalence of jaundice (70%), weight loss (83.3%), and anorexia (90%) was markedly higher in GBC patients. Compared to other studies [1, 2, 11, 13], these symptoms were more prevalent in our GBC cohort, indicating advanced disease. The statistically significant differences (p < 0.001) in jaundice, weight loss, anorexia, pruritus, and dark urine highlight their importance as distinguishing features of malignancy. Previous studies [1, 10] have also emphasised that constitutional symptoms and cholestatic features are more pronounced in cancer.

A family history of cancer was more common in cases than in controls (23.3% vs. 6.7%). This aligns with studies by Sinha SR et al. [11], who reported a positive family history in 18% of cases, and Kumar A et al. [1], who reported a positive family history in 20.5% of cases. However, the difference was not statistically significant (p = 0.071). A family history of GBC was reported in four cases and none of the controls, representing a statistically significant difference (p = 0.038). This suggests a possible genetic predisposition, which is supported by a molecular study by Erices JI et al. [14] that highlighted genetic susceptibility to GBC in the Chilean population. Sharma N et al. [15] and Baruah C et al. [16] performed molecular analyses of bile and serum, respectively, in Indian cohorts and emphasised genetic and environmental interactions in disease development.

Only 6.7% of patients had an ECOG score of 0, compared to 18-22% reported in the literature [1], while a higher proportion had ECOG scores of 2 (40%) and 3 (20%). Similar trends were observed by Sinha SR et al. [11], who reported that approximately 35% of patients had ECOG scores ≥2. The higher proportion of advanced ECOG scores in our study reflects late presentation and aggressive disease biology. Previous studies [1, 10] have also highlighted that poor performance status correlates with advanced staging and poor prognosis.

Upon analysing the laboratory parameters, haemoglobin levels in the carcinoma group were significantly lower than those in controls (10.2 vs. 12.4 g/dL), consistent with the 10.8 g/dL reported by Bind MK et al. [12] and 11.1 g/dL reported by Sinha SR et al. [11], reflecting anaemia of chronic disease. The NLR was significantly higher (3.8) compared to the 3.1 reported by Kumar A et al. [1] and 2.9 reported by Sarangi Y and Kumar A [9]. Similarly, the PLR was 178.4, exceeding the range of 150-165 reported in studies by Sinha S et al. [11] and Agrawal S et al. [17]. These elevated inflammatory markers are supported by studies such as that by Goydos JS et al. [18]. Additionally, systemic inflammation has been linked to tumour progression and poor prognosis by Utsumi M et al. [19].

Total bilirubin levels were significantly higher in the GBC group than in controls (3.8 vs. 0.9 mg/dL). This was higher than the 3.1 mg/dL reported by Sinha SR et al. [11] and 2.8 mg/dL reported by Kumar A et al. [1]. Serum albumin levels were 2.9 g/dL, which was lower than the 3.2 g/dL reported by Kumar A et al. [1]. This finding is supported by Utsumi M et al. [19], who demonstrated that hypoalbuminaemia is associated with poor prognosis in GBC.

CA 19-9 levels were significantly elevated in carcinoma patients compared to controls (214.6 vs. 22.4 U/mL), which was higher than the 180 U/mL reported by Bind MK et al. [12] and 165 U/mL reported by Sinha SR et al. [11]. A meta-analysis by Zhou X [13] also reported elevated CA 19-9 levels, with a pooled sensitivity ranging from 72% to 85%. CEA levels were 8.6 ng/mL in our study, which was higher than the 6.8 ng/mL reported by Sinha SR et al. [11] and 7.2 ng/mL reported by Kumar A et al. [1]. Similarly, CA 125 levels of 58.4 U/mL exceeded the 45-50 U/mL reported in earlier studies [12, 17]. Molecular biomarkers such as miRNA-21 (ΔCt = 3.82 ± 1.24) and miRNA-155 (ΔCt = 2.94 ± 1.08) were significantly elevated in controls, which is consistent with findings by Srivastava P et al. [20], who reported similar miRNA-21 levels (ΔCt = 3.2 ± 1.1), and Erices JI et al. [14], who highlighted the role of genetic biomarkers. Studies on exosomal miRNAs [21, 22] also support their high diagnostic potential. All biomarkers showed strong statistical significance (p < 0.001), supporting their diagnostic accuracy. These findings also support the role of combined biomarker panels in the accurate diagnosis of GBC, as emphasised in recent literature [9, 23]. The inclusion of miRNA markers in our study provides additional diagnostic strength and aligns with emerging trends in non-invasive cancer diagnostics.

GB wall thickening >4 mm was observed in 93.3% of the GBC group, which was higher than the 85% reported by Patkar S et al. [24] and 82.4% reported by Kumar A et al. [1]. Similarly, an intraluminal mass was detected in 73.3% of our cases, exceeding the 65.2% reported in radiological studies [25] and 68% reported by Sinha SR et al. [11]. Gallstones were present in 60% of patients in the malignancy group, which was lower than the 70-85% association reported by Rawla P et al. [10] and 72.5% reported by Kumar A et al. [1]. Porcelain GB was observed in 20% of cases compared to the 10-15% reported in earlier studies [10, 26]. Pericholecystic collection (26.7%) and liver metastasis (33.3%) were also more frequent than the 20% and 25%, respectively, reported by Patkar S et al. [24]. Biliary dilatation (53.3%) was more frequent than the 45% reported by Sinha SR et al. [11]. The statistically significant differences (p < 0.001) highlight that USG findings such as wall thickening, mass lesions, and biliary dilatation are strong indicators of malignancy. Previous studies [1, 24] have emphasised that USG remains the first-line imaging modality, but its diagnostic accuracy increases when combined with clinical and biomarker data.

An enhancing GB mass was observed in 93.3% of GBC patients, which was higher than the 85.6% reported by Patkar S et al. [24] and 88% reported by Kumar A et al. [1]. Liver infiltration was seen in 90% of patients, exceeding the 45% reported by Sinha SR et al. [11] and 48.2% reported in other cohorts [25]. Nodal involvement in our study was 76.6%, which was higher than the 52% reported by Kumar A et al. [1] and 55.5% reported by Sinha SR et al. [11]. Specifically, porta hepatis nodes (53.3%) and retroperitoneal nodes (33.3%) were more frequently involved compared to the 40% and 20%, respectively, reported in earlier studies [24]. Vascular encasement (30%) was also more frequent than the 25-28% reported in previous literature [25]. Distant metastasis was observed in 33.3% of patients, higher than the 18-22% reported by Rawla P et al. [10] and Kumar A et al. [1]. The statistically significant differences (p < 0.001) highlight the role of CECT in staging and operability assessment. Previous studies [24, 25] have highlighted that CECT is superior to USG in detecting nodal involvement and vascular invasion.

The majority of patients were in advanced stages, with T3 (23.3%) and T4 (33.3%) comprising 56.7% of cases. This was similar to the 55-60% reported by Kumar A et al. [1] and 58.4% reported by Sinha SR et al. [11]. Nodal involvement was present in 70% (N1 + N2), which was higher than the 55-60% reported in previous literature [1, 25]. Metastatic disease (M1) was observed in 33.3% of patients, higher than the 18-20% reported by Rawla P et al. [10]. Overall staging showed that stage III and IV disease constituted 70% of cases, compared to the 60-65% reported in earlier Indian studies [1]. This underscores the need for early diagnostic biomarkers, as emphasised in recent molecular studies [9, 23].

Moderately differentiated tumours were the most common (43.3%), which was comparable to reports by Sinha SR et al. [11] and Kumar A et al. [1]. However, the proportion of poorly differentiated tumours in our study (36.7%) was higher than the 28-32% reported in earlier studies [10], indicating more aggressive disease. Previous molecular studies [14, 15] have shown that poorly differentiated tumours are associated with a higher mutation burden and worse prognosis. Adenocarcinoma was the predominant histological type (80%), consistent with the 85-90% reported in the literature [1, 27]. Lymphovascular invasion was present in 53.3%, exceeding the 45% reported by Sinha SR et al. [11], while perineural invasion (46.7%) was also more frequent than the 35-40% reported in earlier cohorts [1]. Negative resection margins (R0) were achieved in 88.9% of surgical patients, comparable to the 80-85% reported by Agrawal S et al. [17].

CA 19-9 levels increased from 98.4 U/mL in stages I-II to 214.8 U/mL in stage III and 384.2 U/mL in stage IV (p < 0.001), which was higher than the stage-wise increase reported by Sinha SR et al. [11], where values ranged from approximately 85 U/mL in early stages to 300 U/mL in advanced stages. Similarly, Agrawal S et al. [17] reported CA 19-9 levels increasing from nearly 90 U/mL to 280 U/mL across the stages. CEA levels in our study increased from 4.2 ng/mL (stages I-II) to 15.2 ng/mL (stage IV), which was higher than the 3.5-12.8 ng/mL reported by Kumar A et al. [1] and 4.0-13.5 ng/mL reported by Bind MK et al. [12]. CA 125 levels also demonstrated a progressive increase (32.6-94.2 U/mL), exceeding the 28-75 U/mL reported in previous studies [11, 17]. Notably, the ΔCt values of miRNA-21 decreased significantly from 5.96 to 1.92, compared to the range of 4.8-1.5 reported by Srivastava P et al. [20] and other molecular studies [21, 22]. These findings indicate a strong correlation between biomarker levels and tumour burden and are supported by previous studies [9, 23], which have emphasised that biomarker escalation reflects tumour progression.

The present study demonstrated that CA 19-9 had an AUC of 0.892, with a sensitivity of 86.7% and specificity of 83.3%, which was higher than the pooled sensitivity of 72-82% reported by Zhou X [13] and comparable to the reported specificity of 80-85%. Similarly, Bind MK et al. [12] reported a sensitivity of 78% and specificity of 82%. CEA showed a sensitivity of 73.3% and specificity of 76.7% in our study, comparable to the 65-72% sensitivity reported by Sinha SR et al. [11] and Kumar A et al. [1]. CA 125 demonstrated a sensitivity of 76.7% and specificity of 80%, which was slightly higher than the 70-75% reported in earlier studies [17]. Importantly, miRNA-21 showed the highest diagnostic performance among the individual biomarkers, with an AUC of 0.914, sensitivity of 90%, and specificity of 86.7%, which was similar to the 85-88% sensitivity reported by Srivastava P et al. [20] and 82-86% reported in other molecular studies [21, 22]. The combined panel (CA 19-9 + miRNA-21) achieved an AUC of 0.956, with 93.3% sensitivity and 90% specificity, which was similar to the 88-92% accuracy reported in recent biomarker studies [9, 23]. These findings indicate that combined biomarkers improve diagnostic accuracy. Prospective, randomised trials with larger sample sizes are needed to validate the use of a combined biomarker panel.

CA 19-9 levels were significantly higher in unresectable cases (264.4 vs. 98.4 U/mL), consistent with findings by Agrawal S et al. [17] and Sinha SR et al. [11]. Similarly, CEA levels (10.5 vs. 4.2 ng/mL) were higher, consistent with those reported by Kumar A et al. [1]. miRNA-21 (ΔCt values) showed a marked change (4.53 in resectable vs. 1.92 in unresectable cases), comparable to that reported by Erices JI et al. [14]. This indicates higher miRNA expression in unresectable or advanced cases. Previous studies [17, 23] have demonstrated that elevated CA 19-9 and CEA levels correlate with poor resectability and survival. These results also support the use of biomarkers for surgical decision-making and prognostic stratification.

Only 30% of carcinoma patients underwent curative surgery compared to 93.3% of controls, which was lower than the 40-45% reported by Dai JW et al. [28] and 38% reported by Kumar A et al. [1]. Palliative chemotherapy was required in 46.7% of patients, higher than the 35-40% reported in earlier studies [28]. Radical cholecystectomy accounted for 30% of cases, comparable to the 15-25% reported in surgical series [17]. The use of gemcitabine + cisplatin (46.7%) aligns with standard regimens reported in recent oncology literature [28]. Open surgery (22.2%) and radical procedures (66.7% of surgical cases) were more frequent than laparoscopic approaches. Previous studies [1, 17] have also shown that minimally invasive surgery is less feasible in advanced cancer. The statistically significant differences (p < 0.001) highlight that most carcinoma patients undergo palliative management.

A total of 26.7% of carcinoma patients showed a treatment response, which was lower than the 35-40% response rates reported by Dai JW et al. [28]. Progressive disease was observed in 36.7% of patients, higher than the 25-30% reported in earlier studies [1]. Mortality in our study was 46.7%, which was higher than the 35-40% reported by Rawla P et al. [10] and Kumar A et al. [1]. Median survival was 8.4 months, lower than the 10-12 months reported in previous studies [17, 28]. The proportion of patients alive at follow-up (40%) was lower than the 55-60% reported in earlier cohorts [1]. These findings reflect aggressive disease and late-stage presentation. Previous studies [10, 28] have emphasised that survival is closely linked to the stage at diagnosis.

The present study demonstrated a strong positive correlation between CA 19-9 and histological grade (r = 0.642), which was higher than the r = 0.58 reported by Sinha SR et al. [11]. Similarly, CEA showed a correlation (r = 0.534), comparable to the 0.48 reported by Kumar A et al. [1]. The CA 125 correlation (r = 0.486) was slightly higher than the 0.42 reported in earlier studies [17]. miRNA-21 showed a strong negative correlation (r = -0.718), exceeding that reported by Srivastava P et al. [20] and supporting its role as a molecular marker. Albumin also showed a negative correlation (r = -0.594), consistent with findings by Utsumi M et al. [19], who reported a similar inverse relationship. Bilirubin showed a weak correlation (r = 0.312), comparable to previous studies [11]. The statistically significant correlations (p < 0.001) indicate that higher biomarker levels are associated with poorer differentiation and aggressive tumour biology. Previous molecular studies [14, 29] have also highlighted the role of genetic and inflammatory markers in tumour progression.

CA 19-9 levels were higher in carcinoma patients without gallstones (238.6 vs. 198.4 U/mL). This trend was comparable to findings by Sinha SR et al. [11], who reported higher CA 19-9 levels (~220 vs. 180 U/mL) in non-gallstone-associated cancers. CEA levels (9.8 vs. 7.8 ng/mL) were higher in patients without gallstones, similar to those reported by Bind MK et al. [12]. CA 125 levels also followed a similar pattern (64.8 vs. 54.2 U/mL), consistent with earlier studies [17]. The ΔCt values of miRNA-21 were also higher in patients with gallstones (4.12 vs. 3.62), which aligns with findings by Srivastava P et al. [20]. Studies on metabolomic and genetic differences [16, 29] have also indicated distinct biological pathways in gallstone-associated and non-gallstone-associated cancers. The lack of statistical significance across all biomarkers (p > 0.05) suggests that, while numerical differences exist, gallstone status does not significantly influence biomarker levels. These results reinforce that biomarkers are reliable diagnostic tools irrespective of gallstone status and can be universally applied in clinical practice.

Limitations

The present study was conducted at a single tertiary care centre with a small sample size (the majority of cases were also in advanced stages), which may limit the generalisability of the findings and reduce the strength of subgroup analyses. The case-control observational design of the study restricts the ability to establish a definitive causal relationship between biomarker levels and GBC. The availability and cost of advanced molecular diagnostic techniques may limit their routine applicability in resource-limited environments. The study did not perform serial monitoring of biomarkers and therefore could not assess their role in the diagnosis of early-stage GBC, disease progression, treatment response, and long-term prognosis. Potential confounding factors, such as nutritional status, coexisting inflammatory conditions, liver dysfunction, and variability in tumour biology, could have influenced biomarker levels. Therefore, large-scale, multicentre studies with longer follow-up are needed to validate these findings, standardise biomarker cut-off values, and explore their role in early diagnosis, prognosis, treatment monitoring, and survival prediction.

## Conclusions

GBC is a silent killer characterised by its aggressive nature, ability to mimic benign pathology, and dismal outcomes. The use of non-invasive molecular biomarkers in the accurate diagnosis of GBC has the potential to improve disease management in the future, leading to higher resectability rates and better survival outcomes. Our study establishes the role of these markers in differentiating advanced disease from benign GB pathology. Their role in early-stage disease remains exploratory. The use of a combined panel of molecular biomarkers and conventional tumour markers is a promising diagnostic approach that requires validation in larger, prospective studies.
